# Solvent extraction and gas chromatography–mass spectrometric determination of probable carcinogen 1,4-dioxane in cosmetic products

**DOI:** 10.1038/s41598-020-62149-x

**Published:** 2020-03-23

**Authors:** Ibrahim Hotan Alsohaimi, Mohammad Rizwan Khan, Hazim Mohammed Ali, Mohammad Azam, Ahmed Moid Alammari

**Affiliations:** 1 Chemistry Department, College of Science, Jouf University, Sakaka, Saudi Arabia; 2 Department of Chemistry, College of Science, King Saud University, Riyadh, 11451 Saudi Arabia; 3 Forensic Chemistry Department, Forensic Medicine Authority, Cairo, Egypt

**Keywords:** Environmental impact, Quality of life

## Abstract

In the present work, a method based on solvent extraction and gas chromatography–mass spectrometry (GC-MS) has been validated for the determination of 1,4-dioxane in cosmetics. Various solvents including ethyl acetate, hexane, methanol, dichloromethane and acetone have been used for the extraction of 1,4-dioxane, among them the ethyl acetate was found to be the most efficient extracting solvent. This method has offered excellent quality parameters for instance linearity (*R*^2^ > 0.9991), limit of detection (LOD, 0.00065-0.00091 µg/mL), limit of quantification (LOQ, 0.00217–0.00304 µg/mL) and, precision intra-day (1.65–2.60%, n = 5) and inter-day (0.16–0.32%, n = 5) in terms of relative standard deviation (RSD%). A total of thirty-nine cosmetic samples of different brands and origin have been studied. Among them, the 1,4-dioxane was found in twenty-three samples (FB_1_-FB_7_, MC_1_-MC_4_, MC_6_-MC_8_, HS_3_, HS_5_, BL_1_-BL_3_, BL_5_ and PLD_1_-PLD_3_) at the levels between 0.15 µg/mL and 9.92 µg/mL, whereas in sixteen samples (MC_5_, HS_1_, HS_2_, SG_1_-SG_5_, BL_4_ and HP_1_- HP_6_) was found to be not detected. The recovery values were achieved between 93% and 99% in both low and high level of spiked samples. In comparison to the traditional analytical techniques, the proposed method was found to be very sensitive and cost-effective for the routine analysis of 1,4-dioxane at low concentration in cosmetics.

## Introduction

1,4-Dioxane is a synthetic compound mainly used as solvent in industrial based products for instance paints, waxes, polishes, inks and varnishes. It is also present in cosmetic formulations and personal care products as dispersants, lathering agents and emulsifiers^1–3^. In the meantime, the non-ionic surfactants have occupied an extensive proportion of global surfactant consumption, and are being used in cosmetics and associated products^4,5^. The non-ionic surfactants can be categorized into three groups with polyethylene oxide, polyhydric alcohol, and poly(ethylene/propylene) oxide. In the course of the ethoxylation reaction procedure, 1,4-dioxane can be produced as a by-product through the dimerization of ethylene oxide^2,5,6^. 1,4-dioxane has also been identified in various water samples^7–13^, and since 1978, the occurrence of 1,4-dioxane as a water pollutant is recognized in the United States^8^.

Based on sufficient toxicological information of 1,4-dioxane carcinogenicity in animals, the International Agency for Research on Cancer (IARC)^14^ and Integrated Risk Information System (IRIS)^15^ have classified 1,4-dioxane as a *probable human carcinogen*. Regarding to toxicity profile, the Environmental Protection Agency (EPA) has identified a chronic oral reference dose of 0.03 mg/kg/day based on the toxicity of kidney and liver in mice and rats^15,16^. The toxicity investigations have identified that the 1,4-dioxane is associated to the toxicity of internal organs and enhance the formation of tumors in such kinds of animals^17,18^. Because of all gathered 1,4-dioxane toxicological data, its presence in various matrices including cosmetics, food and environmental^5,6,19,20^, formerly many analytical procedures have been developed. Primarily the 1,4-dioxane has been identified in water samples using various analytical procedures for instance solid-phase extraction/gas chromatography-mass spectrometry (SPE-GC–MS)^6,9,21^, headspace solid-phase microextraction (headspace-SPME)-GC–MS^22^. Besides, the 1,4-dioxane has also been found in many cosmetics (shampoo^2,5,23–25^, conditioner^5^, cleanser^5^, dishwashing liquid^5,23^, liquid soap^5,23,25^, day cream^5,24,25^, after-shave emulsion^24^, moisturizing lotion^5,24,25^, sun cream^24,25^, baby lotion^25^, bath foam^25^, cleansing milk^25^, after-shave balm^25^ and hair lotion^25^) using different analytical techniques for instance SPE–GC–FID^2^, headspace–SPME–GC–MS^5^, headspace–gas chromatography–mass spectrometry (headspace–GC–MS)^23^, SPE–high-performance liquid chromatography–UV detector (HPLC–UV)^24^ and SPE–GC–MS^26^. In previous literatures, the 1,4-dioxane contamination in cosmetics was frequently identified and required additional considerations to end the use of it for health concerns. The developed methods, nevertheless, undergoes from various shortcomings for instance the necessity for extensive sample pretreatment including expensive and time taking. The sensitivity, recovery and precision were also low in these earlier techniques^5,24–27^. Therefore, a simple, precise and sensitive analytical procedure was required for the identification of 1,4-dioxane in cosmetics.

The objective of the present work was to develop an analytical method based on low-cost solvent extraction and GC–MS for the analysis of 1,4-dioxane in a broad range of commercially available cosmetics. The method has been demonstrated to be proficient of measuring 1,4-dioxane at trace level lower than the recommendations value^28,29^. The overall high throughput including sensitivity, recovery and precision presented by the proposed technique can be an advantage for this kind of investigation.

## Results and discussion

### Optimization of solvent extraction method

**Figure 1:**
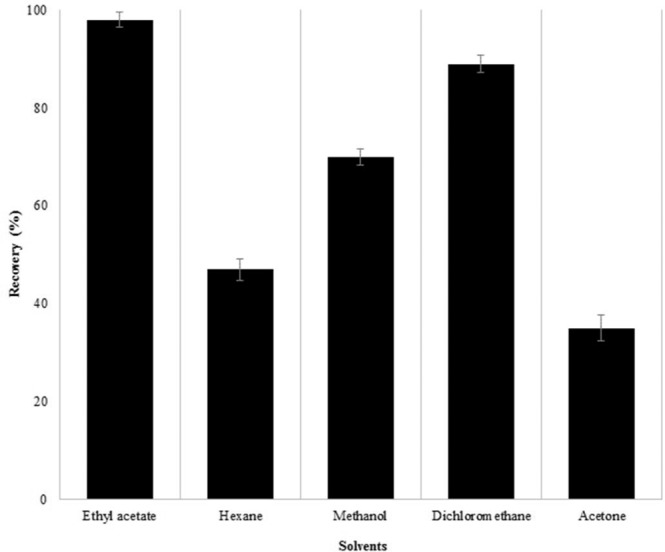
Measured recovery values using pure extracting organic solvents, recovery rates was based on spike-and-recovery method. The standard error bar is the estimated values of the standard deviation (s) of means of analyzed samples.

The assay of 1,4-dioxane in cosmetics was carried out by solvent extraction and GC–MS methods, respectively. Relating to the optimization of extracting solvents, initially, water was chosen as extracting solvent but 1,4-dioxane was found to have higher solubility due to the low octanol/water partition coefficient, and thus it was very challenging to extract 1,4-dioxane in aqueous medium using solvent extraction method. To sort out this problem, we have selected various pure extracting solvents for instance ethyl acetate, hexane, methanol, dichloromethane and acetone, and attempted to vary many parameters to extract 1,4-dioxane from cosmetics. The extraction procedures have been described as a sample extraction procedure section, the identical extraction procedures were applied for each extracting solvent. Among the tested extracting solvents, the ethyl acetate, hexane, methanol, dichloromethane and acetone have offered 1,4-dioxane recovery values of 99%, 47%, 70%, 89% and 35%, respectively. Among them, the ethyl acetate was found to be the most efficient extracting solvent where the 1,4-dioxane recovery value reaches up to 99%, nonetheless, the acetone offered the least 1,4-dioxane recovery value (35%). In previously reported study, the authors had applied combined liquid–liquid extraction (dichloromethane as extracting solvent) and GC–MS method^30^ for the identification of 1,4-dioxane in water samples, obtained excellent recovery up to 95%. Comparatively, the sample matrix was somewhat dissimilar but the recovery values were found to be in good agreement with the values obtained in current study. Nevertheless, the chlorinated solvents should be avoided to use as extracting solvent due to the environmental concern. Thus, the ethyl acetate was selected as an extracting solvent for the extraction of 1,4-dioxane in cosmetics. The enhancement, in the extraction of the target compound, can be because of the diverse composition of cosmetics. The effect of extracting solvents on recovery values has been demonstrated in Fig. 1. The Fig. 1 comprises a standard error bar of the mean which is the estimated values of the standard deviation (s) of means of analyzed samples, this is calculated using formula s/√(n), where n corresponds to the number of replicates (n = 5).

### Method validation

**Figure 2:**
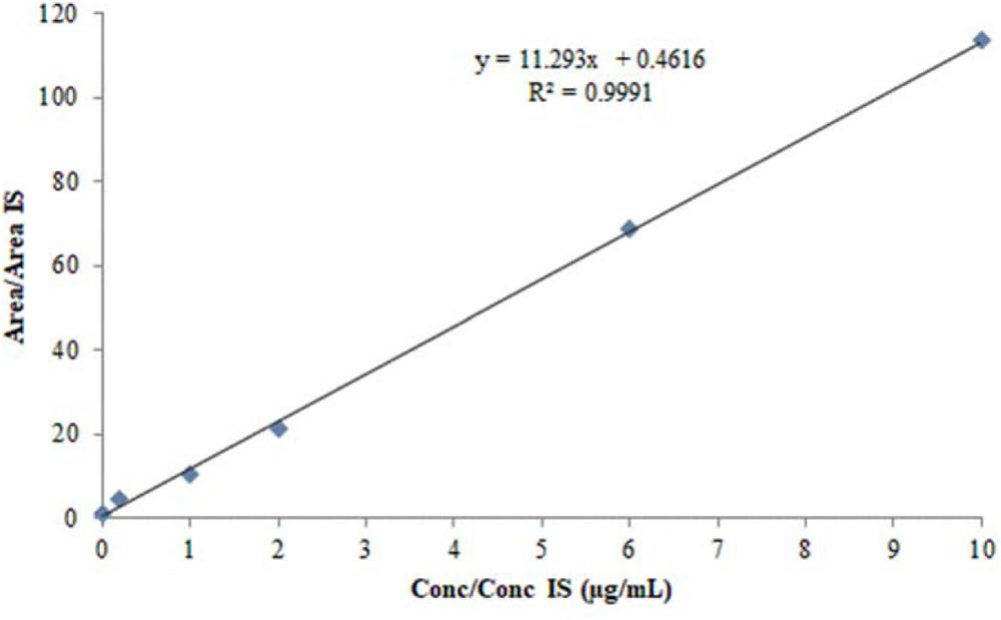
Calibration curve of 1,4-dioxane, obtained from the measured ratio values of the peak area of 1,4-dioxane to the peak area of cyclohexanone (IS) vs. ratio of the 1,4-dioxane and cyclohexanone (IS) concentrations.

In order to assess the accurateness of the proposed solvent extraction-GC/MS procedure for the identification of 1,4-dioxane in cosmetics, the linearity, limits of detection (LOD, signal-to-noise ratio of 3:1) and limits of quantification (LOQ, signal-to-noise ratio of 10:1), precision (intra-day and inter-day) and accuracy were studied. The linearity of the method was investigated at six concentrations between 0.005 µg/mL and 50 µg/mL. This higher range was chosen due to 1,4-dioxane which is expected to be detected at low level (ng/L) to higher level µg/mL^2^. The linearity of the system was investigated by constructing calibration plot of the ratio of the peak area value of target compound 1,4-dioxane to the peak area value of cyclohexanone vs. ratio of the amounts of 1,4-dioxane and cyclohexanone. The linear range, slope, intercept and correlation coefficient (*R*^2^) of 1,4-dioxane have been illustrated in Fig. 2.

Sensitivity in terms of LOD and LOQ of the method was measured from cosmetic samples (MC_5_, HS_1_, HS_2_, HS_4_, SG_1_, SG_2_, SG_3_, SG_4_, SG_5_, BL_4_, HP_1_, HP_2_, HP_3_, HP_4_, HP_5_) free from 1,4-dioxane. These samples were spiked with low amounts (0.001 µg/mL) of 1,4-dioxane at the beginning of the extraction process. The achieved results have been illustrated in the Table 1. LOD of the solvent extraction-GC/MS in these cosmetic samples were achieved between 0.00065 µg/mL to 0.00091 µg/mL, whereas the LOQ obtained was between 0.00217 µg/mL and 0.00304 µg/mL (Table 1). The achieved outcomes show that the offered solvent extraction-GC/MS technique is appropriate for the determination of 1,4-dioxane in different kinds of cosmetic samples even at very low concentrations. The system precision was assessed by the intra-day and inter-day analysis. The intra-day analysis discusses to the determination of five samples injection in the same day whereas inter-day analysis discusses the determination of five samples injection over three consecutive days at low (0.3 µg/mL) and high (1.0 µg/mL) concentrations. The excellent intra-day (1.65–2.60%, n = 5) and inter-day (0.16–0.32%, n = 5) values in terms of RSD% were achieved. The precision of the system has been presented in Table 2.

**Table 1:** The obtained LOD and LOQ values of 1,4- dioxane in cosmetic samples.

Sample*	LOD	LOQ
MC_5_	0.00065	0.00217
HS_1_	0.00083	0.00278
HS_2_	0.00091	0.00304
HS_4_	0.00084	0.00279
SG_1_	0.00078	0.00259
SG_2_	0.00094	0.00314
SG_3_	0.00087	0.00289
SG_4_	0.00091	0.00304
SG_5_	0.00075	0.00250
BL_4_	0.00077	0.00256
HP_1_	0.00080	0.00268
HP_2_	0.00091	0.00302
HP_3_	0.00088	0.00292
HP_4_	0.00083	0.00277
HP_5_	0.00086	0.00286
HP_6_	0.00088	0.00294

LOD, limit of detection (signal-to-noise, 3:1);

LOQ, limit of quantification (signal-to- noise, 10:1);

*1,4- dioxane was not found in these samples.

**Table 2:** Accuracy and precision of the proposed method for the determination of 1,4-dioxane.

Conc. added (µg/mL)	Intra-day			Inter-day		
	Conc. found^a^ (µg/mL) ± SD	Recovery^b^ (%)	RSD (%)	Conc. found^a^ (µg/mL) ± SD	Recovery^b^ (%)	RSD (%)
0.3	0.296 ± 0.0005	98	1.65	0.280 ± 0.0046	93	2.60
1.0	0.997 ± 0.0260	99	0.16	0.942 ± 0.0029	94	0.32

^a^Mean concentration (n = 5); ^b^mean recovery (n = 5); SD = standard deviation (n = 5); RSD = relative standard deviation.

The outcomes have revealed that the present method can be proposed for the routine analysis of 1,4-dioxane in cosmetic samples at very low concentrations. Until now, a few of literatures relating to the determination of 1,4-dioxane in cosmetics are available. In one of the study, the author has applied combined method based on headspace–GC–MS for the analysis of 1,4-dioxane in cosmetic products comprising polyethoxylated surfactants, found higher LOD value (0.3 µg/mL)^27^. Nevertheless, in another studies, the authors have applied combined headspace-SPME-GC–MS method for the analysis of 1,4-dioxane in nonionic surfactants and cosmetics, and SPE–GC–flame ionization detector (FID) detecting identification of 1,4-dioxane in cosmetic raw materials and finished cosmetic products, respectively, where the authors also detected higher LOD values ranged from 0.06 to 0.51 µg/mL^5^. Alternative method based on SPE-HPLC-UV detector using LiChrospher CH-8 reversed-phase column was also applied for the analysis of 1,4-dioxane in cosmetic samples, where the lowest measurable concentration was found to be 6.5 µg/g^24^. In comparison to the LOD value (0.00065 µg/mL) obtained in our work, the available traditional method offered lower sensitivity including solvent consumption, expensive materials and it was time consuming. As a result, the proposed method have offered advantages over sensitivity, rapidness, faster and inexpensive, and therefore the method could be applied as an advance technique for the routine analysis of 1,4-dioxane at trace level in cosmetics. Furthermore, there are other literatures available about 1,4-dioxane identification in water samples by means of numerous analytical methods for instance SPE-GC–MS^6,10,21^, and headspace–SPME-GC–MS^22^. Relatively, the outcomes achieved in the present work were also found in good agreement with the results obtained in water samples^6,10,22^, and thus the proposed method could also be applied for the analysis of 1,4-dioxane in water samples.

### Application to cosmetic samples

**Figure 3:**
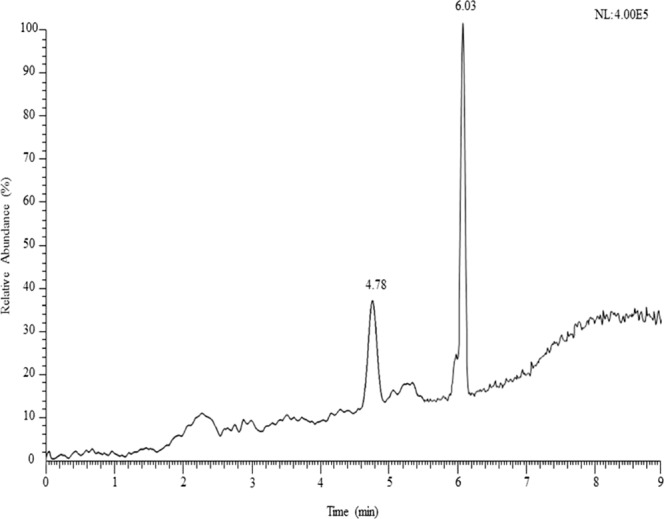
GC-MS chromatogram of 1,4-dioxane (RT, 4.78) and cyclohexanone (IS, RT, 6.03) of facial and body scrub (FB_3_, Perfect Cosmetics) sample, 1,4-dioxane qualifier ions (*m/z*, 88 and 58) and cyclohexanone qualifier ions (*m/z*, 98 and 55).

The developed procedure in the present work was practically applied to examine the1,4-dioxane in commercially available cosmetic samples in Saudi Arabia. A total of thirty-nine cosmetic samples of different brand and origin have been investigated. The achieved results including concentrations and recovery values of 1,4-dioxane in cosmetic samples have been presented in Table 3. The 1,4-dioxane was found in most of the analyzed samples at levels between 0.15 to 9.92 µg/mL whereas in some samples (MC_5_, HS_1_, HS_2_, SG_1_-SG_5_, BL_4_ and HP_1_-HP_6_) the 1,4-dioxane found to be not detected. Recovery values were achieved from 93–98% at low level spiked concentrations and 95 to 99% at high level spiked concentrations. Relatively lower recovery values were obtained in low level spiked concentrations, the cause may be due to the samples complexity and higher loss during the sample extraction procedure. The amounts of 1,4-dioxane in facial and body scrub were obtained between 0.29 µg/mL and 9.92 µg/mL whereas in moisturizing cream (not detected-0.88 µg/mL), hair shampoo (not detected-0.23 µg/mL), shower gel (not detected), body lotion (not detected-0.16 µg/mL), hand soap (not detected) and powder laundry detergent (0.15 µg/mL and 0.21 µg/mL) were obtained. Among the analyzed samples, facial and body scrub, and powder laundry detergent produced 1,4-dioxane in all brand with highest concentration up to 9.92 µg/mL particularly in FB_4_ sample, however, the lowest concentration was obtained in PLD_1_, PLD_2_ and PLD_5_ samples up to 0.15 µg/mL. In shower gel and hand soap sample the 1,4-dioxane has not been identified in any brands. To demonstrate the outcomes, the GC-MS chromatogram of 1,4-dioxane (retention time (RT), 4.78) and cyclohexanone (RT, 6.03) of facial and body scrub (FB_3_, Perfect Cosmetics) sample have been displayed in Fig. 3.

**Table 3:** Levels of 1,4-dioxane and recovery values in finished cosmetic products.

Sample Type	Sample Code	Brand	Origin	Before addition, 1,4-dioxane (µg/mL) ± SD	Added 1,4-dioxane, low level (µg/mL)	After addition, 1,4-dioxane, low level (µg/mL) ± SD	Recovery (%), low level	Added 1,4-dioxane, high level (µg/mL)	After addition, 1,4-dioxane, high level (µg/mL) ± SD	Recovery (%), high level
Facial and body scrub	FB_1_	Bonus	China	0.29 ± 0.01	0.05	0.3375 ± 0.05	95	0.3	0.5824 ± 0.03	97
	FB_2_	BERORN BEAUTY	UAE	0.34 ± 0.02	0.05	0.3880 ± 0.05	96	0.3	0.6312 ± 0.05	97
	FB_3_	Perfect Cosmetics	UAE	0.56 ± 0.02	0.05	0.6085 ± 0.04	97	0.5	1.0480 ± 0.08	98
	FB_4_	Reo	UK	9.92 ± 0.11	0.05	9.9690 ± 0.03	98	10	19.8012 ± 0.20	99
	FB_5_	Facial Scrub	UAE	0.55 ± 0.02	0.05	0.5978 ± 0.04	96	0.5	1.0381 ± 0.08	98
	FB_6_	Berries FACIAL and BODY SCRUB	UAE	0.34 ± 0.01	0.05	0.3870 ± 0.05	94	0.3	0.6322 ± 0.03	97
	FB_7_	Hams of Natural	UAE	0.32 ± 0.01	0.05	0.3677 ± 0.05	95	0.3	0.6121 ± 0.03	97
Moisturizing Cream	MC_1_	LAMSAT HARIER	UAE	0.23 ± 0.01	0.05	0.2770 ± 0.05	94	0.2	0.4234 ± 0.04	97
	MC_2_	BAZA NANCY STAR	China	0.71 ± 0.02	0.05	0.7580 ± 0.03	96	0.7	1.3943 ± 0.10	98
	MC_3_	Fantastic	UAE	0.88 ± 0.03	0.05	0.9281 ± 0.03	96	0.9	1.7655 ± 0.10	98
	MC_4_	bio glow PAPAYA	UAE	0.16 ± 0.01	0.05	0.2065 ± 0.05	93	0.2	0.3543 ± 0.02	97
	MC_5_	Oud Abiyad	UAE	ND	0.05	0.0465 ± 0.06	93	0.2	0.1920 ± 0.01	96
	MC_6_	BODY Cream	UAE	0.63 ± 0.01	0.05	0.6780 ± 0.03	96	0.6	1.2189 ± 0.07	98
	MC_7_	icare TOTAL BODY CARE	India	0.18 ± 0.01	0.05	0.2271 ± 0.04	94	0.2	0.3748 ± 0.03	97
	MC_8_	Body Butter COCOA	UAE	0.21 ± 0.01	0.05	0.2565 ± 0.04	93	0.2	0.4045 ± 0.03	97
Hair Shampoo	HS_1_	BAZA NANCY STAR	China	ND	0.05	0.0465 ± 0.06	93	0.2	0.1910 ± 0.02	96
	HS_2_	Perfect Cosmetics	UK	ND	0.05	0.0464 ± 0.06	93	0.2	0.1900 ± 0.02	95
	HS_3_	BASAMAD	China	0.23 ± 0.01	0.05	0.2771 ± 0.03	94	0.2	0.4241 ± 0.04	97
	HS_4_	SHAMPOO Henna	UAE	ND	0.05	0.0464 ± 0.06	93	0.2	0.1910 ± 0.02	96
	HS_5_	EVIPEIS COCONUT	China	0.16 ± 0.01	0.05	0.2068 ± 0.04	94	0.2	0.3536 ± 0.03	97
Shower Gel	SG_1_	Amalfi Classic gel	Spain	ND	0.05	0.0463 ± 0.06	93	0.2	0.1910 ± 0.02	96
	SG_2_	AQUA VERA COSMETICS	Turkey	ND	0.05	0.0465 ± 0.05	93	0.2	0.1900 ± 0.02	95
	SG_3_	ALYANS	Turkey	ND	0.05	0.0468 ± 0.05	94	0.2	0.1910 ± 0.02	96
	SG_4_	BELUX	Turkey	ND	0.05	0.0469 ± 0.06	94	0.2	0.1910 ± 0.02	96
	SG_5_	SHOWER GEL Strawberry	Turkey	ND	0.05	0.0465 ± 0.05	93	0.2	0.1910 ± 0.02	96
Body Lotion	BL_1_	NOURSHING body lotion	UAE	0.15 ± 0.01	0.05	0.1968 ± 0.05	94	0.2	0.3432 ± 0.03	97
	BL_2_	iCARE TOTAL body care	UAE	0.16 ± 0.01	0.05	0.2075 ± 0.04	95	0.2	0.3540 ± 0.03	97
	BL_3_	Papaya Extract	Indonesia	0.15 ± 0.01	0.05	0.1970 ± 0.05	94	0.2	0.3436 ± 0.03	97
	BL_4_	FREE CARE Natural Wheat	China	ND	0.05	0.0469 ± 0.06	94	0.2	0.1930 ± 0.02	97
	BL_5_	FREE CARE Romantic	China	0.16 ± 0.01	0.05	0.2065 ± 0.05	93	0.2	0.3543 ± 0.03	97
Hand Soap	HP_1_	Lifebuoy	KSA	ND	0.05	0.0468 ± 0.05	94	0.2	0.1910 ± 0.02	96
	HP_2_	Soph	Turkey	ND	0.05	0.0465 ± 0.06	93	0.2	0.1930 ± 0.02	97
	HP_3_	LUX	KSA	ND	0.05	0.0466 ± 0.06	93	0.2	0.1910 ± 0.02	96
	HP_4_	U&U	KSA	ND	0.05	0.0467 ± 0.05	93	0.2	0.1910 ± 0.02	96
	HP_5_	GENTO	KSA	ND	0.05	0.0468 ± 0.06	94	0.2	0.1900 ± 0.02	95
	HP_6_	Impra	KSA	ND	0.05	0.0465 ± 0.05	93	0.2	0.1910 ± 0.02	96
Laundry detergent	PLD_1_	BONUX	KSA	0.15 ± 0.01	0.05	0.1973 ± 0.04	95	0.2	0.3444 ± 0.03	97
	PLD_2_	ARIEL	KSA	0.15 ± 0.01	0.05	0.1973 ± 0.04	95	0.2	0.3448 ± 0.03	97
	PLD_3_	Prino	KSA	0.21 ± 0.01	0.05	0.2578 ± 0.03	96	0.2	0.4065 ± 0.04	98

SD: Standard deviation (n = 3), ND: not detected, UK: United Kingdom, UAE: United Arab Emirates, KSA: Kingdom of Saudi Arabia

It can be seen that the excellent resolution, symmetry and sensitivity was attained in the analysis, no tailing and interfering peaks were perceived at the same retention time of the studied analyte in cosmetic samples. Fuh and his coworkers have studied the presence of 1,4-dioxane in shampoo, liquid soap and dish washing detergent samples. The 1,4-dioxane was detected in shampoo samples at concentrations ranged from 11.5 to 41.1 µg/mL, whereas in liquid soap and dish washing detergent, the 1,4-dioxane was detected at concentrations ranged from 7.8 to 6.5 µg/mL^5^. These values were found in good agreements with the values obtained in the current study. In another work, Rastogi (1990) identified 1,4-dioxane in 82% of the studied cosmetic samples at amounts ranged from 0.3 to 96 µg/mL, and 85% of the dish washing samples contained 1,4-dioxane between 1.8 µg/mL and 65 µg/mL^27^. The variation in the concentrations was might be due to the different composition of the products, brand and origin. The results obtained from the current study are the substantial data source related to the presence of 1,4-dioxane in cosmetic samples of various brand and origin that are commercially available in the Saudi Arabian markets. The 1,4-dioxane could be primarily originated from the contaminations of nonionic surfactants through production. The cosmetic products are being worthy of further concerns, as they are extensively applied in our everyday existence. Table 4 displays the comparison of analyzed 1,4-dioxane in cosmetics from Saudi Arabia and with the earlier reported data. The LOD value in the present study was significantly enhanced as compared to those LOD values obtained in traditional extraction methods. Furthermore, the current method has also offered shorter analysis time, excellent precision and recovery values. According to the European Union directives on the safety of cosmetics^31,32^, 1,4-dioxane must not be applied in their preparations. Nevertheless, 1,4-dioxane was remains identified in cosmetic products in Saudi Arabia. Thus, the observing and reducing the amount of 1,4-dioxane in cosmetic products required additional concerns to encounter greater necessities of guidelines to protect user’s health.

**Table 4:** Comparison of results with the previously developed method for the analysis of 1,4-dioxane in cosmetic products.

Extraction method	Sample treatment time (min)	Determination method	1,4-dioxane (µg/mL)	RSD (%)	Recovery (%)	LOD (µg/mL)	References
Solvent extraction	19	GC–MS	0.280–0.997	0.16–2.60	93–99	0.00065	Current study
SPE	30	GC	10	19.9	70–80	0.5	^26^
Headspace	960	GC–MS	0.3–96	9.1	92–94	0.3	^27^
SPE	40	GC–MS	–	4.3	–	3	^33^
Ultrasound–assisted extraction and SPE	49	GC–MS/MS	0.23–15.30	<5	84–108%	0.2	^34^
SPME	10	GC	0–40.1	1.4	98.6	0.3	^5^
SPME	10	GC–MS	0–41	3.2	96	0.06	^5^
Headspace single–drop microextraction	10–30	GC	2.4–201	7.2	84	0.4	^35^

## Conclusions

A low cost, sensitive and reliable procedure based on solvent extraction and GC/MS has been developed for the determination of 1,4-dioxane in various cosmetic samples. Many solvents (methanol, hexane, dichloromethane and ethyl acetate) have been applied for the extraction of 1,4-dioxane, among them ethyl acetate was found to be the most efficient extracting solvent. A total of thirty-nine cosmetic samples of different brands and origin have been investigated, 1,4-dioxane was detected in most of the analyzed samples at concentrations ranged from 0.15 to 9.92 µg/mL, while in some samples (MC_5_, HS_1_, HS_2_, SG_1_- SG_5_, BL_4_ and HP_1_-HP_6_) 1,4-dioxane found to be not detected. The excellent quality parameters including recovery values (up to 99%) were achieved. The applied technique illustrates insignificant matrix effect and the lowest sample pretreatment needed deprived of losing target analyte. The inexpensive solvent extraction procedure implemented through the experimentation is a substantial benefit since during the sampling procedure it reduces the analysis period and the possibility of negligible losses of the studied compound and thus assists to offer enhanced method performance. The obtained results including excellent quality parameters and negligible matrix influences have made possible the applicability of the offered procedure as a new method for the routine examination 1,4-dioxane in cosmetic products.

## Materials and Methods

### Chemicals and reagents

The solvents and chemicals used in the current investigation were of analytical or liquid chromatography grade, obtained from Merck (Darmstadt, Germany). 1,4-dioxane and cyclohexanone were supplied from Sigma Aldrich (St. Louis, USA). Sodium sulfate anhydrous was purchased from Merck (Darmstadt, Germany). For sampling procedure, ultrapure water was produced using water filtration system (Milli–Q, Millipore Corporation, Bedford, USA). The stock standard solution of 1,4-dioxane was prepared in methanol at level 1000 µg/mL and applied for additional dilution methods. The IS solution was prepared in methanol at concentration 5 µg/mL and added to calibration solutions and samples during analysis. To verify the linearity (*R*^2^) of the procedure, 1,4-dioxane standard at various levels (0.005–50 µg/mL) were made using weight by weight. 1,4-dioxane standard solutions and cosmetics were filtered through syringe filter (0.22 μm) (PTFE, Macherey-Nagel GmbH, Düren, Germany) before being injected into the GC-MS system.

### Sample extraction procedure

Cosmetic samples of various brands and origin were purchased from cosmetic and pharmacy shops located in Saudi Arabia. Cosmetic samples were refrigerated between 2–4 °C and investigated within one week to evade any microbial contamination. For the extraction of 1,4-dioxane from cosmetic samples, 1 g of cosmetic sample was accurately weighed in a 40 mL glass tube followed by the addition of 5 mL ethyl acetate. The sample solution was thoroughly mixed by means of vortex mixer (Jeio Tech, Seoul, South Korea) for 1 min. Afterward, the sample was moved to ultrasonic bath for sonication (10 min) at room temperature, sample centrifugation was carried out by HERMLE, model Z32 HK centrifuge system (Wehingen, Germany) at 6000 rpm for 8 min. The sample supernatant was taken out and moved to separating funnel followed by the addition of sodium sulfate to extract water contents. Finally, the sample was collected in a new glass tube and solvent was evaporated by means of nitrogen gas. Then, the extract of the sample was redissolved with 1 mL methanol (containing IS, 5 µg/mL) standard followed by mixing using vortex system. The 1 mL sample solution was filtered by means of PTFE syringe filter (0.22 μm) and injected into the GC-MS system. The sample injection volume was 1 µL to the splitless mode.

To evaluate the effectiveness of the extraction method and preventing the effect of matrix influences on retention time, peak intensity and shape, the quantification of 1,4-dioxane was carried out by a standard addition method comprising two non-spiked samples at zero levels and three spiked samples at 50% (0.1 µg/mL, values representing the increase of 1,4-dioxane in the sample after spiking), 100% (0.2 µg/mL) and 500% (1.0 µg/mL). The samples spiking was performed at the beginning of each extraction procedure. Samples were examined in triplicates, and the statistical analysis was carried out using ANOVA method.

The LOD and LOQ of the procedure were assessed in cosmetic samples (MC_5_, HS_1_, HS_2_, HS_4_, SG_1_, SG_2_, SG_3_, SG_4_, SG_5_, BL_4_, HP_1_, HP_2_, HP_3_, HP_4_, HP_5_) free from 1,4-dioxane. These samples were spiked with low amounts (0.001 µg/mL) of 1,4-dioxane before being analyzed by the optimized method. The recovery values of 1,4-dioxane were estimated at low and high concentrations in cosmetic samples, achieved from the added and found 1,4-dioxane concentrations. The quality control parameters of the system were also studied in each sample lot to confirm that the contamination of the samples did not come out and the sensitivity of the system was steady throughout the study.

### Gas chromatography-mass spectrometric conditions

GC–MS analysis was carried out on a gas chromatograph (TRACETM 1310 GC) equipped with single quadrupole mass spectrometer (ISQLT) and auto-sampler unit model AI/AS1310 (Thermo Scientific, Waltham, USA). Separation of 1,4-dioxane was carried out on analytical Rxi-624Sil MS column with dimension 60 m, 0.53 mm I.D., 3.0 µm thickness (Restek, USA). The applied temperature program was started at 40 °C for 3 min, then rising to 240 °C at a rate of 50 °C/min (10 min). The flow-rate of helium (carrier gas) was controlled to get 100 kPa. The temperatures of injector, transfer line and ion source were adjusted to 250 °C. Ion source was established in electron ionization mode with 70 eV. The sample injection volume was 1 µL and operated to the splitless mode. The identification of the target analyte was performed using a mass spectrometer system. The data acquisition, data reporting, method setup and data processing were monitored using Xcalibur 3.1 software (Thermo Scientific, Waltham, USA).

**Figure 4:**
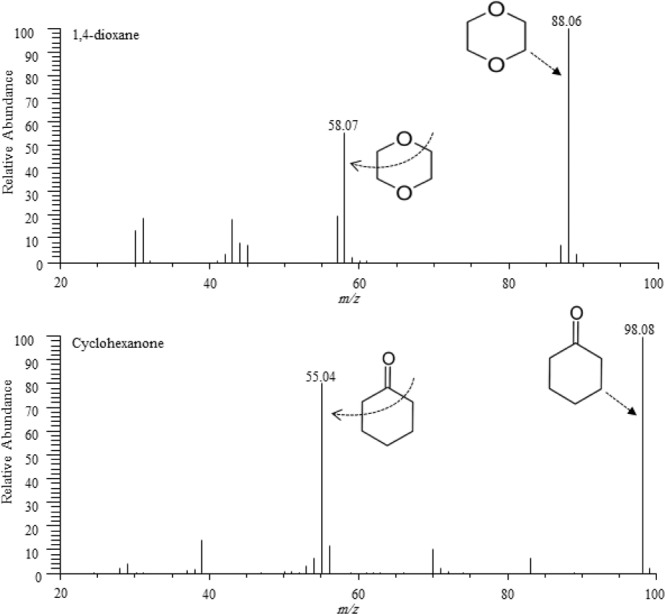
Mass spectrum and fragmentation patterns of 1,4-dioxane (*m/z*, 88 58) and cyclohexanone (IS, *m/z*, 98 55) in the studied samples.

To identify the target analytes, mass spectral database search methods was applied from National Institute of Standards and Technology (NIST, Gaithersburg, MD, USA). Gas chromatography-mass spectrometric analysis was carried out on a gas chromatograph (TRACETM 1310 GC) equipped with single quadrupole mass spectrometer (ISQLT) and auto-sampler unit model AI/AS1310 (Thermo Scientific, Waltham, USA). To identify the target analytes, mass spectral database search methods was applied from NIST. Prior to the analysis, the ionization mass spectra scan of 1,4-dioxane and cyclohexanone (IS) was carried out at concentration of 5 μg/mL. The electron ionization mass spectra scan was ranged from *m/z*, 20–300, and quantification was based on selected ion monitoring (SIM). The representing ions were *m/z*, 88 and 58 (1,4-dioxane) and *m/z*, 98 and 55 (cyclohexanone), respectively. The base peak of 1,4-dioxane and cyclohexanone was examined, and the particular qualifier ions were used as the established ions for further analysis. The obtained mass spectrum and fragmentation pattern of 1,4-dioxane (*m/z*, 88 and 58) and cyclohexanone (*m/z*, 98 and 55) have been displayed in Fig. 4.

## Supplementary information

Supplementary Information.
