# Supplementary material for: Solvent extraction and gas chromatography–mass spectrometric determination of probable carcinogen 1,4-dioxane in cosmetic products
Source: Sci Rep. 2020 Mar 23;10:5214. doi: 10.1038/s41598-020-62149-x (PMC7090059; doi:10.1038/s41598-020-62149-x)
Supplement: Supplementary file 1 — Supplementary Information. [file 41598_2020_62149_MOESM1_ESM.pdf]

Solvent extraction and gas chromatography–mass spectrometric determination of probable carcinogen 1,4-dioxane in cosmetic products

Ibrahim Hotan Alsohaimi, Mohammad Rizwan Khan, Hazim Mohammed Ali, Mohammad Azam, Ahmed Moid Alammari

Sample Type	Sample Code	Brand	Origin	Before addition, 1,4-dioxane (µg/mL)	Added 1,4-dioxane, low level (µg/mL)	Total Conc., (µg/mL)	After addition, 1,4-dioxane, low level (µg/mL)	Recovery (%), low level	Added 1,4-dioxane, high level (µg/mL)	Total Conc., (µg/mL)	After addition, 1,4-dioxane, high level (µg/mL)	Recovery (%), high level
Facial and body scrub	FB_1	Bonus	China	0.29	0.05	0.34	0.3375	95	0.3	0.59	0.5824	97
	FB_2	BERORN BEAUTY	UAE	0.34	0.05	0.39	0.3880	96	0.3	0.64	0.6312	97
	FB_3	Perfect Cosmetics	UAE	0.56	0.05	0.61	0.6085	97	0.5	1.06	1.0480	98
	FB_4	Reo	UK	9.92	0.05	9.97	9.9690	98	10	19.92	19.8012	99
	FB_5	Facial Scrub	UAE	0.55	0.05	0.6	0.5978	96	0.5	1.05	1.0381	98
	FB_6	Berries FACIAL and BODY SCRUB	UAE	0.34	0.05	0.39	0.3870	94	0.3	0.64	0.6322	97
	FB_7	Hams of Natural	UAE	0.32	0.05	0.37	0.3677	95	0.3	0.62	0.6121	97
Moisturizing Cream	MC_1	LAMSAT HARIER	UAE	0.23	0.05	0.28	0.2770	94	0.2	0.43	0.4234	97
	MC_2	BAZA NANCY STAR	China	0.71	0.05	0.76	0.7580	96	0.7	1.41	1.3945	98
	MC_3	Fantastic	UAE	0.88	0.05	0.93	0.9281	96	0.9	1.78	1.7655	98
	MC_4	bio glow PAPAYA	UAE	0.16	0.05	0.21	0.2065	93	0.2	0.36	0.3543	97
	MC_5	Oud Abiyad	UAE	0.00	0.05	0.05	0.0465	93	0.2	0.20	0.1920	96
	MC_6	BODY Cream	UAE	0.63	0.05	0.68	0.6780	96	0.6	1.23	1.2189	98
	MC_7	icare TOTAL BODY CARE	India	0.18	0.05	0.23	0.2271	94	0.2	0.38	0.3748	97
	MC_8	Body Butter COCOA	UAE	0.21	0.05	0.26	0.2565	93	0.2	0.41	0.4045	97
Hair Shampoo	HS_1	BAZA NANCY STAR	China	0.00	0.05	0.05	0.0465	93	0.2	0.20	0.1910	96
	HS_2	Perfect Cosmetics	UK	0.00	0.05	0.05	0.0464	93	0.2	0.20	0.1900	95
	HS_3	BASAMAD	China	0.23	0.05	0.28	0.2771	94	0.2	0.43	0.4241	97
	HS_4	SHAMPOO Henna	UAE	0.00	0.05	0.05	0.0464	93	0.2	0.20	0.1910	96
	HS_5	EVIPEIS COCONUT	China	0.16	0.05	0.21	0.2068	94	0.2	0.36	0.3536	97
Shower Gel	SG_1	Amalfi Classic gel	Spain	0.00	0.05	0.05	0.0463	93	0.2	0.20	0.1910	96
	SG_2	AQUA VERA COSMETICS	Turkey	0.00	0.05	0.05	0.0465	93	0.2	0.20	0.1900	95
	SG_3	ALYANS	Turkey	0.00	0.05	0.05	0.0468	94	0.2	0.20	0.1910	96
	SG_4	BELUX	Turkey	0.00	0.05	0.05	0.0469	94	0.2	0.20	0.1910	96
	SG_5	SHOWER GEL Strawberry	Turkey	0.00	0.05	0.05	0.0465	93	0.2	0.20	0.1910	96
Body Lotion	BL_1	NOURSHING body lotion	UAE	0.15	0.05	0.20	0.1968	94	0.2	0.35	0.3432	97
	BL_2	iCARE TOTAL body care	UAE	0.16	0.05	0.21	0.2075	95	0.2	0.36	0.3540	97
	BL_3	Papaya Extract	Indonesia	0.15	0.05	0.20	0.1970	94	0.2	0.35	0.3436	97
	BL_4	FREE CARE Natural Wheat	China	0.00	0.05	0.05	0.0469	94	0.2	0.20	0.1930	97
	BL_5	FREE CARE Romantic	China	0.16	0.05	0.21	0.2065	93	0.2	0.36	0.3543	97
Hand Soap	HP_1	Lifebuoy	KSA	0.00	0.05	0.05	0.0468	94	0.2	0.20	0.1910	96
	HP_2	Soph	Turkey	0.00	0.05	0.05	0.0465	93	0.2	0.20	0.1930	97
	HP_3	LUX	KSA	0.00	0.05	0.05	0.0466	93	0.2	0.20	0.1910	96
	HP_4	U&U	KSA	0.00	0.05	0.05	0.0467	93	0.2	0.20	0.1910	96
	HP_5	GENTO	KSA	0.00	0.05	0.05	0.0468	94	0.2	0.20	0.1900	95
	HP_6	Impra	KSA	0.00	0.05	0.05	0.0465	93	0.2	0.20	0.1910	96
Laundry detergent	PLD_1	BONUX	KSA	0.15	0.05	0.20	0.1973	95	0.2	0.35	0.3444	97
	PLD_2	ARIEL	KSA	0.15	0.05	0.20	0.1973	95	0.2	0.35	0.3448	97
	PLD_3	Prino	KSA	0.21	0.05	0.26	0.2578	96	0.2	0.41	0.4065	98
